# Non-Point Source Pollution Simulation and Best Management Practices Analysis Based on Control Units in Northern China

**DOI:** 10.3390/ijerph17030868

**Published:** 2020-01-30

**Authors:** Yang Ding, Fei Dong, Jinyong Zhao, Wenqi Peng, Quchang Chen, Bing Ma

**Affiliations:** 1 State Key Laboratory of Simulation and Regulation of Water Cycle in River Basin, Beijing 100038, China; iwhrdy@163.com (Y.D.); dongfei@iwhr.com (F.D.); pwq@iwhr.com (W.P.); chenqch@iwhr.com (Q.C.); bingma123@163.com (B.M.); 2 China Institute of Water Resources and Hydropower Research, Beijing 100038, China

**Keywords:** control unit, non-point source pollution, SWAT, Best Management Practice

## Abstract

Non-point source (NPS) pollution simulation in control units can identify critical pollution source areas and make Best Management Practices (BMPs) more effective for the responsible parties. In this study, the control unit division method is introduced, and the spatial and temporal distribution characteristics of NPS pollution in the Guishui River Basin of Northern China are analyzed using the Soil Water Assessment Tool (SWAT) model. In addition, five BMP scenarios were designed for environmental and cost-benefit analyses. The results show that the loss of total nitrogen (TN) and total phosphorus (TP) is concentrated in the rainy season, and the loss of TN and TP is mainly distributed in the middle and lower reaches of the main stream of the Guishui River. This area accounts for 22.34% of the basin area. The vegetated filter strips (VFS) scenario had the best environmental benefits with average TN and TP reduction efficiencies of 63.4% and 62.6%, respectively. The Grassed Waterway was the most cost-effective scenario measure, cost-benefit (CE) values of TN and TP were 1798.13 g/€ and 601.56 g/€. Generally, research on NPS pollution using control units can more clearly identify the critical source areas of pollution than other methods, and provides technical support for watershed management decision makers.

## 1. Introduction

At present, most river and lake ecosystems worldwide have encountered problems such as water pollution and degradation of ecological functions, that have seriously affected the sustainable development of economic societies [1]. A survey by the United States Environmental Protection Agency showed that non-point source (NPS) pollution caused ~60% of rivers and lakes water quality to be substandard [2]. Surveys in the United States, Japan, and other countries show that even if all point source pollution achieved zero emissions, the river water, lake water, and sea water quality compliance rates would only be 65%, 42%, and 78%, respectively. Eutrophication of lakes and reservoirs mainly comes from NPS pollution [3]. In recent years, point source pollution has been gradually controlled, and it was found that the main cause of the deterioration of water environmental quality is the increasing NPS, especially NPS pollution caused by human agricultural activities [4,5]. The composition of pollution sources is currently rapidly changing in China, and the proportion of NPS pollution load is gradually increasing [6]. The uncertainty in NPS pollution emissions and pathways has caused NPS to harm agricultural production, water resources, watershed hydrological processes, and the habitat of aquatic organisms [7]. NPS pollution affects the quality of the environment on which humans depend and can directly or indirectly affect public health [8,9].

In the area of basin water environment management, the main purpose of dividing control units is to deconvolve complex basin water environmental problems into each control unit, so that specific water basin water environmental management measures and policies can be effectively implemented to improve the water quality of the river basin [10]. The concept of the control unit was originally derived from the Total Maximum Daily Loads (TMDL) technical guidelines in the United States. It is understood that if the problem in the water body originates from the lower reaches of the basin, such as lakes and reservoirs, the entire target water body is regarded as a TMDL control unit. However, if the problem water body is distributed throughout the watershed, the latter is divided into several sub-watersheds according to the catchment area, and each sub-watershed is a control unit for research purposes [11].

The United States agencies usually study and manage the pollution in control units taking the basin as the control unit and the control unit’s water quality as the goal. The concept of China’s control unit was developed in researching water environmental capacity and total amount control technology during the “Sixth Five-Year Plan” and “Seventh Five-Year Plan”, and the concept of three-level management of the planning area, control area, and control unit was gradually proposed [12]. However, this type of control unit does not have a clear responsibility body, which makes planning management measures challenging to implement efficiently.

The soil and water assessment tool (SWAT) is a long-period distributed watershed hydrological model, which can predict the production and pollution of different regions in the basin under different soil types, land use types, and management measures. It has gradually become an indispensable tool in water resources and water environmental protection management planning and is commonly used to assess the long-term impact of land management models on water flows, sediment, and agricultural nutrients in complex watersheds [13]. With the application and development of model research, the SWAT model has gradually become an important tool for simulating runoff processes, NPS pollution loads, spatio-temporal characteristics, and critical source areas and evaluating different measures at the basin scale [14]. Best Management Practices (BMPs) were a series of measures proposed by the United States in the mid-1970s to generate environmentally beneficial natural processes such as hydrology, soil erosion, ecology, and nutrient cycling in river basins, to avoid pollution in water environments of the river basin caused by agricultural production [15,16,17]. SWAT models were proposed to evaluate the effects of BMPs. Panagopoulos et al. developed an efficient and user-friendly decision support tool to determine the best location for agricultural BMPs and trade-offs between multiple targets to economically and effectively control diffuse pollution across a river basin [18,19]. However, the SWAT model pollution load output results are based on the sub-basin or hydrologic response unit (HRU) as a unit, and only subsequently the critical source areas of the pollution source in the basin are identified. BMPs are usually established based on critical source areas, which only consider the “quantity” of the pollution load, that is, they are only located in heavily polluted areas. If the areas include more than two administrative areas, they will not be conducive to implementing the measures to the responsible body.

In view of this problem, this study introduces a set of control unit division methods, and according to these methods, the control units of the Guishui River Basin in Northern China are divided. The Guishui River Basin is located in the Yanqing District of Beijing and is located in the Beijing-Tianjin-Hebei eco-economic development zone. In 2022, the Yanqing District will host the World Winter Olympics; thus, higher requirements are imposed on the quality of the water environment. In this study, we used the SWAT model to analyze the distribution characteristics of NPS pollution in the Guishui River Basin based on the control unit, and established five BMP scenarios to evaluate the environmental and cost benefits of each scenario. Based on the conclusions of this study, the critical source area of NPS pollution is located in the control unit, and management decision makers can adopt BMPs for the critical source area to provide technical support for management decision makers in identifying and regulating NPS pollution in the basin.

## 2. Materials and Methods 

### 2.1. Study Area

**Figure 1:**
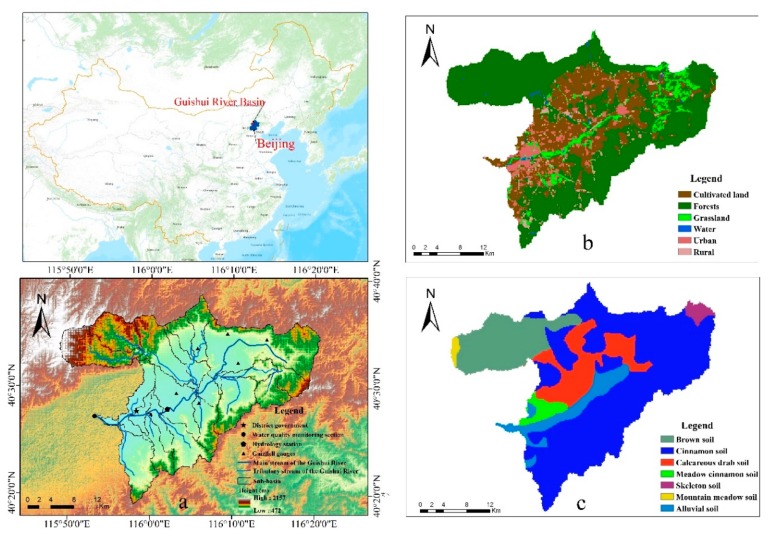
Location of the Guishui River watershed in China.

The Guishui River Basin is located in Yanqing District, Beijing, 74 km from Beijing City. It is the northern gateway of Beijing, and the watershed is located between 115°48′51″ and 116°20′42″ E, and 40°21′50″ and 40°38′40″ N. The total length of the Guishui River is 74.3 km, and its drainage area is 724.6 km^2^. The geographical location of the Guishui River Basin is shown in Figure 1a. The Guishui River Basin is surrounded by mountains on three sides, the climate in the study area is a temperate continental monsoon climate, the average annual precipitation in the whole region is 452 mm, the annual average temperature is 8.7 °C, the maximum temperature is 39 °C, and the minimum temperature is -27.3 °C. The amount of surface water has been changing in the study area for many years, and the inter-annual and intra-annual rainfall changes and distribution are uneven. The rainfall is mainly concentrated between June and September, accounting for ~80% of the annual rainfall. 

The land use in the basin (Figure 1b) is dominated by forest land, accounting for 57.97%, which is concentrated in areas with high terrain on the north and south sides of the Guishui River Basin. The second major use is cultivated land, which accounts for 25.75%, and is concentrated on both sides of the main stream of the Guishui River in the middle of the watershed. Here, loss of agricultural fertilizers (mainly nitrogen fertilizers) can occur through rainfall runoff, which can easily generate NPS pollution. Residential areas account for ~9.63%, of which medium and high-density residential areas are 6.58%, and are mainly concentrated in the central area of Yanqing District downstream of the Guishui River Basin. The main soil types (Figure 1c) in the Guishui River Basin are Cinnamon soil, Brown soil and Calcareous soil. Among them, the area of Cinnamon soil is the largest, accounting for 59.22% of the area of the Guishui River Basin, while 16.29% of the area is Brown soil, and 12.84% is Calcareous brown soil.

### 2.2. Control Unit Division Method

#### 2.2.1. Information Required for Control Unit Division

The data collected in the control unit include: administrative divisions, underlying areas of river basins, river systems, locations of control sections, and sources of pollution:

(1) Administrative Division Information

According to the needs of regional watershed water environment management, the basic unit of administrative division is selected; for example, village-level zoning data are selected for village-level management, and township-level zoning data are selected for township management.

(2) Underlying Surface Information

The underlying data of the basin include digital elevation maps (DEM), types of land use in the basin, and characteristics of specific surface units. 

(3) Source of Pollution

Pollution source information mainly includes the main pollution sources and their distribution in the river basin, as well as the main sewage outlets and their distribution in the river.

#### 2.2.2. Control Unit Division Principle

(1) River Basin Integrity

The principle of river basin integrity means that according to the characteristics of the hydrological cycle of the river basin, the division of control units must ensure the integrity of the river basin and uniformly plan the division scheme of the river basin control unit.

(2) Principle of Determining Land by Water

The control unit is the surface area corresponding to the land and water, and the natural water system is the benchmark for land area division. The land confluence range is determined according to the characteristics of the natural watershed to form a land–water combined unit.

(3) Principle of Completeness of Administrative Division

The boundary of the control unit does not cross the boundary of the administrative district. For the only district or county leading the sewage discharge, the entire district or county is assigned to a certain control unit. For districts and counties that do not have the only dominant drainage destination, districts and counties can be split into different control units.

#### 2.2.3. Control Unit Division

**Figure 2:**
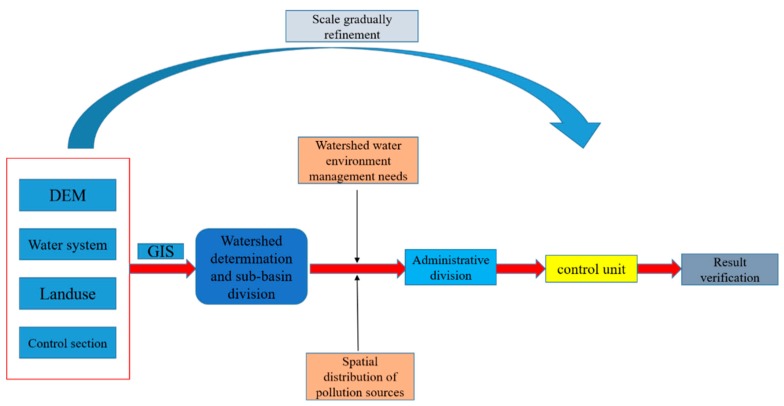
Control unit partitioning step.

The control unit division steps (Figure 2) are as follows:

(1) Related Data Collection

Collect the data required for the division of control units proposed in Section 2.2.1.

(2) Watershed Boundary Identification and Sub-Watershed Division

Based on digital elevation map (DEM) data, hydrological analysis tools in GIS are used to form watershed boundaries. The river basin data are extracted and compared with actual river network water systems. Sub-watersheds are divided by superimposing control sections, land use, and other data. They are spatially continuous, and there is no intersection in the upstream and downstream relationships of the water system. There are obvious differences in the attributes of vegetation and land use among different zones, and different zones show different ecological and hydrological characteristics.

(3) Analysis of Pollution Characteristics in River Basins

Simple analysis of types (industrial, agricultural, domestic) and distribution of pollution sources in the basin, based on the characteristics of the water system and the sub-basin, the topological relationship among point sources and non-point sources in the river basin and their corresponding sewage discharge outlets and tributaries into the river basin is roughly established. It is important that point sources and non-point sources in the same control unit flow into the same tributary tributary or the same main stream section.

(4) Superimposed Administrative Division

Based on the results of small scale partitioning, the control unit is generated by superimposing the results with the data of administrative division and administrative station.

(5) Partition Result Verification

According to the above four steps, the control unit should be connected with the place where the river basin is located and verified by experts. If it meets the needs of the water environment and water ecology management in the river basin, it should be regarded as the final result of the division of the control unit, otherwise, further adjustments should be made. The adjustment is mainly based on the situation of sewage going to and collecting water on the spot, and systematic investigation is made of the hydrology, water environment, administrative division, and social and economic status of the river basin, to make the result of the final division of control unit more reasonable.

### 2.3. SWAT Model

#### 2.3.1. Parameter Calibration Method

The key data collection of the SWAT model in this study is shown in Table 1. Model calibration and validation is done using the sequential uncertainty fitting algorithm (SUFI2) provided by SWAT-CUP [20,21]. This method considers the uncertainty of the data and automatically selects a set of parameters according to certain rules to make the objective function optimal. Various hydrological and water quality parameters were changed to best fit observations within their range [7]. Data from 2014 were used as the preheating period of monthly runoff, data from 2015 and 2016 were used for calibration, and data from 2017 were used for validation. Data from 2014 were used as the preheating period for total nitrogen (TN) and total phosphorus (TP), data from 2015 were used for calibration, and data from 2016 were used for validation. The Nash-Sutcliffe (*E_NS_*) and coefficient of determination (*R*^2^) were used to evaluate the simulation effect of the model [22,23,24], according to the following expressions:

(1) $$E_{NS}=1-\frac{\sum_{i=1}^{n}{\left(O_{i}-P_{i}\right)}^{2}}{\sum_{i=1}^{n}{\left(O_{i}-\bar{O}\right)}^{2}}$$

(2) $$R^{2}={\left[\frac{\sum_{i=1}^{n}\left(O_{i}-\bar{O}\right)\left(P_{i}-\bar{P}\right)}{\sqrt{\sum_{i=1}^{n}{\left(O_{i}-\bar{O}\right)}^{2}}\sqrt{\sum_{i=1}^{n}{\left(P_{i}-\bar{P}\right)}^{2}}}\right]}^{2}$$

where *E_NS_* is the Nash-Sutcliffe efficiency coefficient, *O_i_* is the observation at time *i*, *P_i_* is the analog value at time *i*, $\bar{O}$ is the observed mean, *R*^2^ is the certainty coefficient, and $\bar{P}$ is the predicted mean. When *E_NS_* > 0.75, the simulation effect is considered very good, when 0.50 ≤ *E_NS_* ≤ 0.75, the simulation effect is satisfactory, and when *E_NS_* < 0.50, the simulation effect is not good. When *R*^2^ ≥ 0.85, the simulation effect is very good, when 0.85 > *R*^2^ ≥ 0.60, it indicates that the simulation effect is satisfactory, and when *R*^2^ < 0.60, it indicates that the simulation effect is not good.

#### 2.3.2. Parameters Sensitivity Analysis Result

Analysis of 26 parameters affecting runoff and nitrogen and phosphorus based on the SUFI-2 algorithm in SWAT-CUP. In the parameter sensitivity analysis results, the t-star value gives the degree of sensitivity, the larger the absolute value, the more sensitive it is. The *p*-value determines the significance of sensitivity, the closer it is to 0, the more significant it is. The sensitivity results and parameter values are shown in Table 2. 

**Figure 3:**
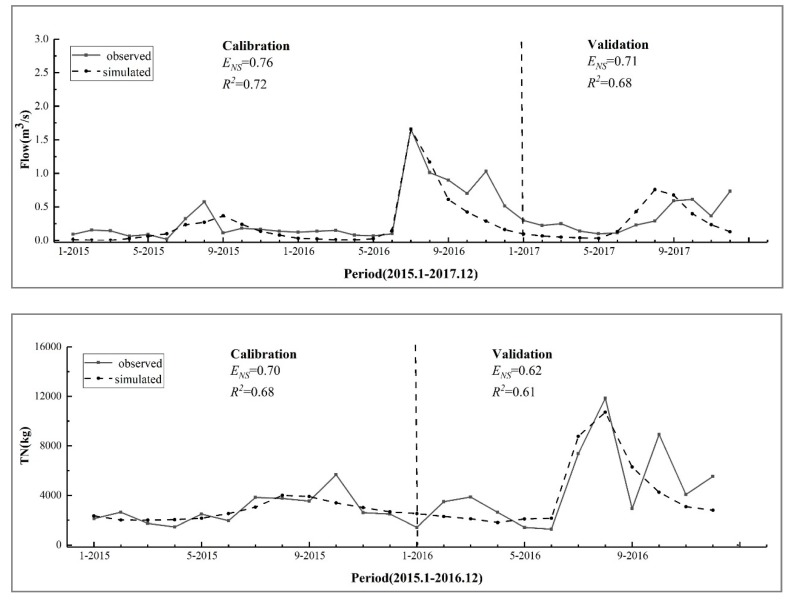

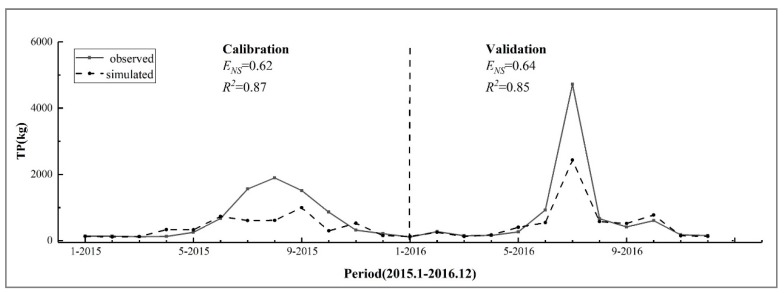
Flow, TN, and TP load curves for the calibration and validation periods.

According to the results, CANMX is the most sensitive parameter in the surface runoff simulation, followed by CN2 and GWQMN. CDN is the most sensitive parameter in the TN simulation, followed by SOL_NO3 and SDNCO. SOL_ORGP is the most sensitive parameter in the TP simulation, followed by P_UPDIS and PHOSKD. Figure 3 shows the monthly fit curves of the flow, TN, and TP during the calibration and verification periods. Among them, Nash-Sutcliffe (*E_NS_*) ≥ 0.5 and *R*^2^ ≥ 0.6, indicating that the model has good applicability.

### 2.4. Best Management Practices

#### 2.4.1. Best Management Practices Setting

According to the current research on BMPs, they can be divided into structural measures and nonstructural measures [26]. Among them, structural measures are mainly to control runoff and sediment to block or change the transport route of pollutants, reduce pollutants from the process, so as to achieve the purpose of NPS pollution control. It is mainly achieved through the use of structural facilities to trap pollutants and circulate them in the sections of pollution sensitive areas, and through physicochemical and biological actions, to separate the direct connection between pollutants and the receiving water body, and block suspended solids and pollutants in the precipitation runoff. Most of the nonstructural measures control the source of pollution and reduce the pollutants entering the receiving water, to reduce the pollutants at the source and achieve the purpose of NPS pollution control. Its principle is to increase agricultural processes and production such as chemical fertilizers, pesticides, and aquaculture waste with the optimized combination of various agricultural management methods, to reduce the pollution to both surface and underground water, the sources of agricultural NPS pollutant and diffusion control, thereby reducing NPS harm to the environment. This research according to the Yanqing District agricultural background and development planning considers the structural measures for Grassed Waterway and Vegetated filter strips (VFS), nonstructural measures for fertilization reduction, and no-tillage. Five scenarios were established (Table 3) to evaluate the nitrogen and phosphorus reduction efficiency and cost-benefit of each scenario in the control unit of the critical source area. 

The Grassed Waterway scenario is a grass-planting channel for conveying field runoff. It has been proven to be an effective treatment measure. Mainly through the plants in the river channel, the flow velocity of field runoff is reduced [27], so that more granular pollutants are deposited [28], thus intercepting and reducing the sediment and pollutants. In this study, grass areas were 0.8 m wide and 0.5 m deep, and were distributed in cultivated land in the Guishui River Basin. 

VFS removes contaminants in a similar way to Grassed Waterways. VFS is a densely vegetated strip used to intercept runoff from upslope sources, effectively reducing pollutants. The VFS width was set at 5 m to assess the reduction and cost of nitrogen and phosphorus loads. The VFS width was manipulated by modifying the parameter FILTERW in SWAT [29]. 

Based on current basin crop management practices, in nonstructural measures, we set fertilization reduction (10% and 20% of the current use amount) and no-tillage to explore the impact of nonstructural measures on nitrogen and phosphorus reduction efficiency.

#### 2.4.2. Best Management Practices Costs

On the premise of meeting the requirements of environmental quality, economic cost becomes an important factor influencing BMP screening. Cost-benefit analysis is an effective BMP optimization method to evaluate various measures according to their costs and benefits. In this study, the difference between the baseline NPS pollution load and the NPS pollution load under various scenarios was taken as the environmental benefit, and the ratio between the environmental benefit and the calculated measure scenario cost was taken as the cost-benefit value (*CE*). *CE* represents the reduction in pollution load per euro; the higher the *CE* value, the better the cost-effectiveness of the measure scenario. Conversely, the lower the *CE* value, the lower the cost-effectiveness of the scenario, that is, the lower the amount of pollutant reduction at the same cost, the less likely it is to be adopted by policy makers [30]. The cost-benefit calculation formula is shown in Equation (3):

(3) $$CE=\frac{LOAD_{BAS}-LOAD_{BMP}}{Cost}$$

where *CE* stands for cost-effectiveness (kg/€); *LOAD_BAS_* and *LOAD_BMP_* represent the NPS production (kg) under the benchmark status quo and the measured scenario, respectively; and Cost represents the cost (€) of the scenario measure deployment.

## 3. Results and Discussion 

### 3.1. Control Unit Division Result

**Figure 4:**
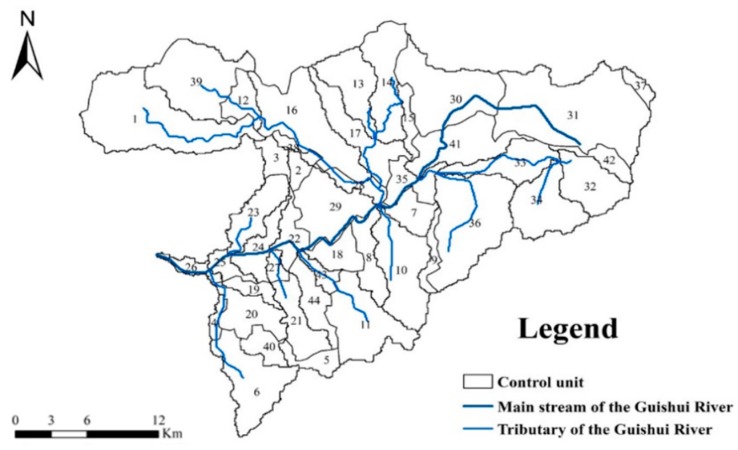
Control unit division results.

According to the division method of control units in Section 2.2, this study divides the Guishui River Basin into 44 control units (Figure 4). The control unit naming and coding adopt the following rules: the name form is “serial number + river + administrative unit”. The specific information of the control unit is shown in Table 4.

### 3.2. Spatial–Temporal Distribution of TN and TP Loads

#### 3.2.1. Time Distribution Character

**Figure 5:**
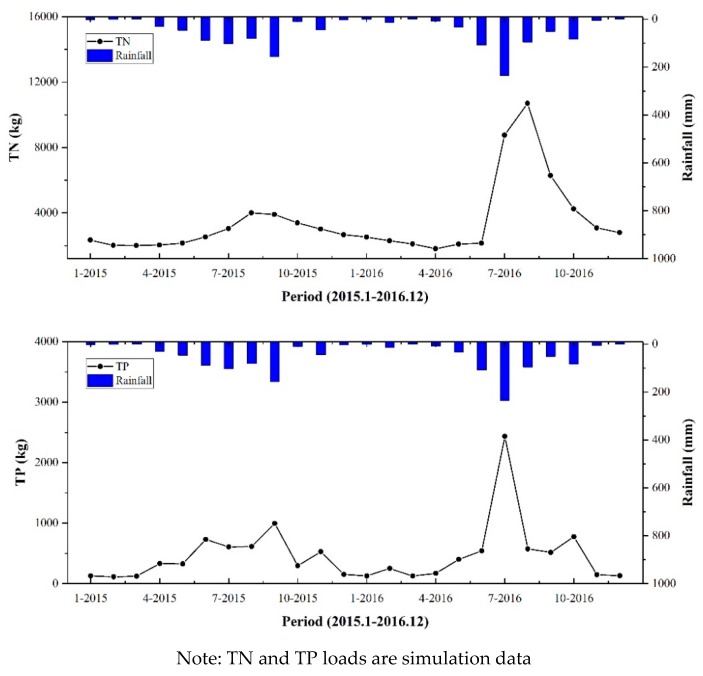
Time distribution of TN and TP loads.

It can be seen from Figure 5 that the TN and TP outputs of the river basin are mainly distributed during the rainy season. The output of TN and TP is also relatively small during periods of light rainfall. As rainfall increases, storms wash away the slope, resulting in increased output. The average rainfall in the flood season (June to September) in 2015 and 2016 accounted for 72.74% of the annual average rainfall, and the output of TN and TP accounted for 50.49% and 62.92% of the annual output, respectively, which shows that rainfall runoff is the main influencing factor for the output of NPS pollution. Most of the non-point source nitrogen and phosphorus pollution is accompanied by rainfall runoff. Large water and sediment volumes in the flood season carry significant pollution load into the river. Meanwhile, the output of pollutants has a certain degree of lag compared to rainfall, and the maximum time of each rainfall does not correspond to the maximum pollutant output.

#### 3.2.2. Spatial Distribution Characteristics

**Figure 6:**
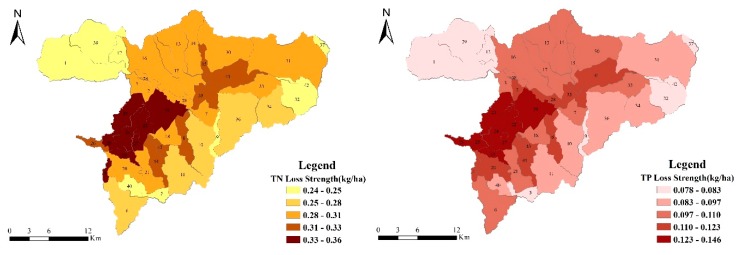
Loss strength of TN and TP in the Guishui River Basin in 2016.

Taking model verification year 2016 as an example to study the spatial distribution characteristics of NPS pollution in the watershed, it can be seen that the difference in pollutant loss strength between different control units is extremely obvious. In 2016, the TN loss strength of the Guishui River Basin was between 0.24 and 0.36 kg/ha, and the TP loss strength was between 0.078 and 0.146 kg/ha. As can be seen from Figure 6, the lower reach of the Guishui River Basin (control units 4, 8, 18, 19, 20, 21, 22, 23, 24, 25, 26, 27, 28, 35, 41, 43, and 44) is a critical source area for pollutant loss.

**Figure 7:**
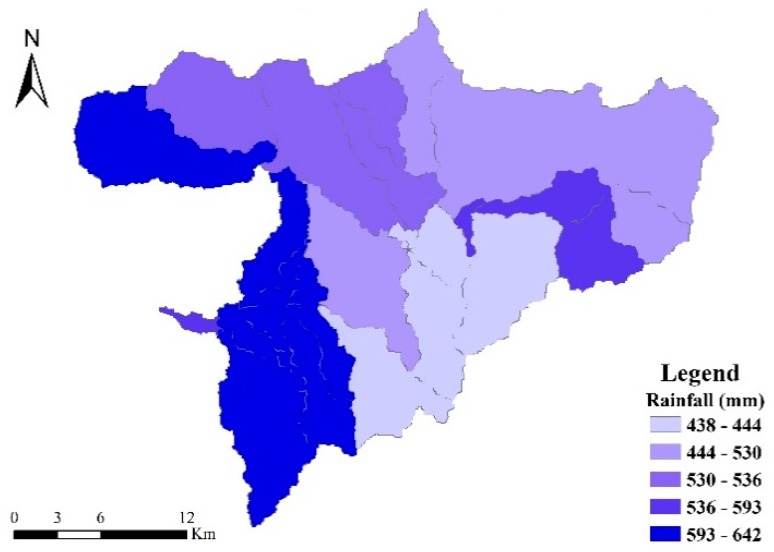
Rainfall distribution in the Guishui River Basin in 2016.

As can be seen from Figure 6, the loss strength distribution of pollutants is roughly the same, the main distribution trend of loss strength is greater in the lower reaches of the basin than in other areas, and it is mainly distributed in the middle and lower reaches of the main stream of the Guishui River. This is mainly because the cultivated land is densely cultivated in this area (Figure 1b), which can easily lead to the loss strength of agricultural fertilizers through the rainfall runoff process. The middle and upper reaches of the watershed were affected by the underlying surface. Most of this area comprises forest land. The high vegetation cover of this land effectively slowed down the soil erosion caused by rain. In addition, the natural growth of root systems is important for nutrition. Substance has a good fixing effect, and the contribution load intensity of forest land is low. Additionally, the distribution of loss strength is roughly the same as the distribution of rainfall (Figure 7), indicating that rainfall is a major factor affecting the strength of pollutant loss.

### 3.3. BMPs Effect Simulation

Frequent human activities are the main reason for the increasing NPS pollution. Appropriate reduction of human impacts and the adoption of a series of structural management measures can alleviate and treat NPS pollution. Based on the spatial distribution characteristics of NPS pollution analyzed in Section 3.2.2 and based on the control unit scale, this study established five scenarios listed in Section 2.4.1 in the watershed. The specific analysis is as follows.

#### 3.3.1. Simulation of Reduction Efficiency

**Figure 8:**
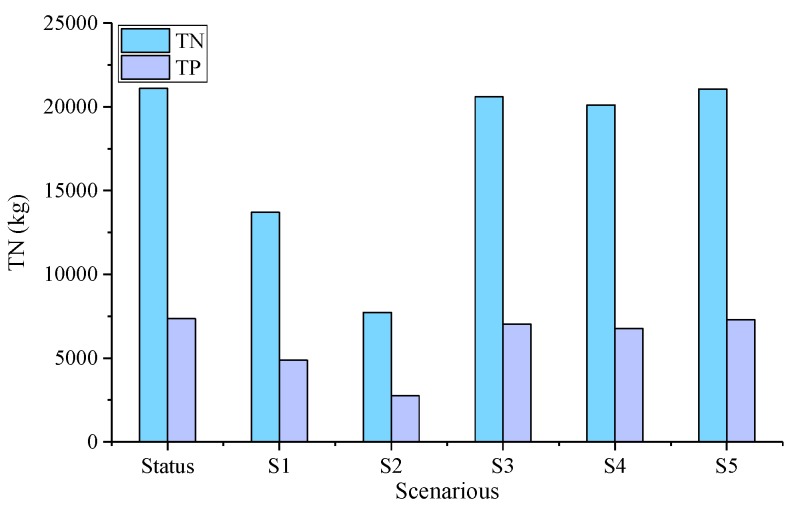
TN and TP loads in each scenario.

The simulation results of five scenarios are shown in Figure 8 and Table 5. The specific scenario analysis is as follows:

(1) Simulation of S1

According to the analysis of the results, the reduction efficiency of TN and TP in Grassed Waterway were 13.2 to 46.3% and 15.6 to 49.6%, and the average reduction efficiency was 35.1% and 33.7%, indicating significant reduction effect. 

(2) Simulation of S2

The reduction efficiencies of TN and TP in VFS were 33.6–76.6% and 43.6–74.2%, and the average reduction efficiencies were 63.4% and 62.6%. S2 is the measure with the highest reduction efficiency out of all five scenarios. 

(3) Simulation of S3 & S4

In S3 and S4, when the fertilizer application amount was reduced by 10%, the reduction efficiencies of TN and TP were 0.3–4.1% and 0.7–12.9%, and the average reduction efficiencies were 2.4% and 4.4%. When the application amount was reduced by 20%, the pollutant reduction efficiency was improved. The reduction efficiencies of TN and TP were 0.5–8.2% and 1–19.4%, and the average reduction efficiencies were 4.7% and 8%. Overall, fertilizer reduction measures had a certain effect on reducing pollutants, but the effect were not particularly significant. This view is consistent with related research [31]. 

(4) Simulation of S5

The results show that the reduction efficiencies of TN and TP were 0–0.9% and 40.2–6.5%, and the average reduction efficiencies were 0.2% and 0.8%. Compared with other scenarios, S5 had the lowest pollutant reduction efficiency.

#### 3.3.2. Cost benefit analysis of BMPs

(1) Cost Accounting

Table 6 shows the costs after setting up the scenario in this study. The total cost of the scenario is 7,004,500 €. The total area of the Grassed Waterway measure layout is 20 ha, the project cost is ~200 €/ha, and the maintenance cost is ~6 €/ha [32]. The VFS measures are arranged around the farmland, at a width of 5 m, and the construction area is 2.5% of the farmland area (473 ha). The buffer forest belt is generally constructed by structural afforestation. The structural cost (including construction costs and other temporary structural costs) is ~350-900 €/ha, based on 600 €/ha, the operating cost is ~15 €/ha per year [32]. The cost of chemical fertilizer reduction is the loss from reduced crop yield after reducing chemical fertilizer application minus the cost savings of reduced chemical fertilizer purchase costs. This study determined the cost of fertilizer reduction through interviews and literature review. According to relevant data, the corn production in the Yanqing area is ~3600 kg/ha, and the purchase price is 0.1 €/kg. The price of urea (calculated as N) is ~0.54 €/kg, and the price of calcium superphosphate (calculated as P_2_O_2_) is ~0.7 €/kg. The measure layout area is the farmland area. According to the literature, the cost of no-tillage measures is ~13.5 €/ha [29].

(2) Cost-benefit analysis

**Figure 9:**
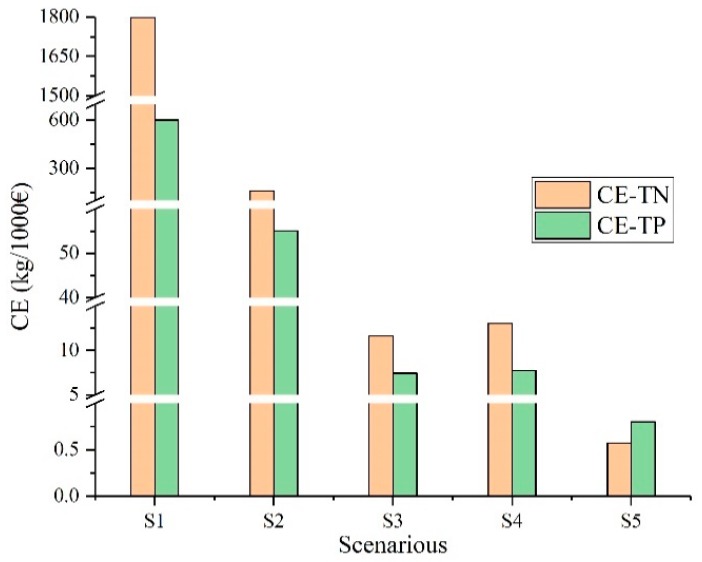
*CE* value in each scenario.

Figure 9 shows the *CE* value of pollutants in each scenario. It can be clearly seen from the figure that the *CE* value of the structural measures (S1 & S2) established in this study is much higher than for the nonstructural measures (S3 & S4 & S5). Among the structural measures, the highest *CE* value is the grass-planting river. The benefits of TN and TP are 1798.13 g/€ and 601.56 g/€. Among nonstructural measures, the *CE* value of no-tillage is the lowest, and the benefits of TN and TP are 0.57 g/€ and 0.8 g/€. It can be seen that the implementation of no-tillage measures in the Guishui River Basin has no obvious effect on pollutant reduction. Although the use of fertilizer reduction measures can reduce pollutants to a certain extent and save fertilizer purchase costs, the resulting reduction in crop production will reduce enthusiasm for the measure. Therefore, managers and decision makers should find the balance between fertilizer reduction and crop yield. From the long-term perspective of *CE* value analysis and watershed management, structural measures are the most effective measures to control pollution load, although this does not mean that only structural measures are used in watershed management. As structural measures occupy a certain amount of land resources, there will be contradictions between governance measures and land resources in future management. Therefore, in the actual governance process, the layout of BMPs needs to take into consideration the land resources, comprehensively consider the local socio-economic level, and ecological environment status, and adopt appropriate BMPs according to the local conditions.

## 4. Conclusions

The novelty of the study is included in the Introduction in terms of mentioning the limitations of the existing approach. However, the SWAT model pollution load output results are based on the sub-basin or hydrologic response unit (HRU) as a unit, and only subsequently the critical source areas of the pollution source in the basin are identified. BMPs are usually established based on critical source areas, which only consider the “quantity” of the pollution load; that is, they are only located in heavily polluted areas. If the areas include more than two administrative areas, they will not be conducive to implementing the measures to the responsible body.

This study systematically applies a well-known methodology to non-point source pollution study for the Guishui River Basin, and tests various methodologies for reducing it. Because of the systematic design of the study, the outcomes are well suited for policy implementation. Based on the control unit scale, the SWAT model was used to analyze the cost-effectiveness of BMP implementation in the Guishui River Basin, and the scenarios and measures were simulated and analyzed from environmental benefits and economic costs perspective:

(1) This study introduces the division method of the river basin control unit. The division of the control unit can effectively implement the measures to the responsible subjects. The control unit analyzes the pollution load of the river basin and clarifies the responsible party.

(2) The SWAT model verified by parameter calibration shows good applicability to the simulation of NPS pollution in the Guishui River Basin. Based on the control unit scale, the spatial and temporal distribution of NPS pollution in the river basin is analyzed. The results show that the time distribution of pollutants in the river basin is extremely uneven, and the pollutant output is mainly distributed in the rainy season. Rainfall runoff is the main influencing factor NPS pollution output. Similarly, there are differences in the spatial distribution of pollutant output. In 2016, the TN loss intensity of the Lushui River Basin was between 0.24 and 0.36 kg/ha, and the TP loss intensity was between 0.078 and 0.146 kg/ha. The lower reach of the Guishui River Basin (control units 4, 8, 18, 19, 20, 21, 22, 23, 24, 25, 26, 27, 28, 35, 41, 43, and 44) is a critical source area for pollutant loss. Therefore, the implementation of governance measures should take into account the spatiotemporal distribution of pollutants, which can be adjusted in time to control units.

(3) This study established five scenarios, including structural and nonstructural measures. The SWAT model was used to simulate the pollutant reduction in each scenario. Simulation results show that the reduction efficiency of structural measures is much higher than that of nonstructural measures, of which S2 pollutants have the highest reduction effect. The average reduction rates of TN and TP were 63.4% and 62.6%, respectively. The S5 pollutant reduction effect was the lowest, and the average reduction rates of TN and TP were only 0.2% and 0.8%. Similarly, under the condition of economic cost, the *CE* value of structural measures set in this study is much higher than nonstructural measures. S3 is the most cost-effective measure, the values for TN and TP are 1798.13 g/€ and 601.56 g/€, Compared with S4, S3 shows a better pollutant reduction rate when using fewer land resources.

In the selection of BMPs in the river basin, in consideration of environmental and economic benefits, factors such as land resources, government development planning and technological level must also be considered, as decision makers are required to take all factors into consideration. Therefore, selecting a variety of effective BMPs reasonably, and providing long-term and effective plans for local environmental protection and sustainable development is key.

In addition, the SWAT model was based on the parameters for the study area only and did not verify those for different areas of the same watershed. This is likely to cause errors in the calculation of runoff and pollution in each sub-watershed. Further research should be conducted on the parameters of each sub-basin in the future.

## Figures and Tables

**Table 1:** Guishui River Basin data.

Type of Data	Resolution/Proportion/Site/Coverage	Period	Format
Digital elevation map	30 × 30 m	2017	GRID
Land use	1:250,000	2017	Arc/Info coverage
Soil type	1:1,000,000	2017	Arc/Info coverage
Meteorological data	Yanqing Station	1959–2017	txt
Rainfall data	Yongning, Xiangying, Liubinbao, Shenjiaying, Jixian, Jingzhuang Station	2014–2017	txt

**Table 2:** Model parameter sensitivity analysis results and parameter values.

Index	Sensitivity	Parameter	t-Star	*p*-Value	Value	File
Flow	1	CANMX	−3.41	0	0.37	.hru
	2	CN2	−3.29	0	65	.mgt
	3	GWQMN	−2.33	0.02	3686.1	.gw
	4	SOL_AWC	2.24	0.03	0.56	.sol
	5	OV_N	−2.14	0.03	0.48	.hru
	6	SMTMP	−1.21	0.23	−5.98	.bsn
	7	SOL_K	−1.11	0.27	110.8	.sol
	8	SMFMN	0.82	0.41	9.38	.bsn
	9	PLAPS	−0.79	0.43	−409.88	.sub
	10	CH_K1	−0.7	0.48	474	.sub
	11	GW_DELAY	−0.66	0.51	132.67	.gw
	12	RCHRG_DP	0.47	0.64	1.22	.gw
	13	TLAPS	−0.37	0.71	−2.09	.sub
TN	1	CDN	3.74	0	0.09	.bsn
	2	SOL_NO_3_	−3.29	0	41.94	.chm
	3	SDNCO	−2.19	0.03	0.54	.bsn
	4	N_UPDIS	1.3	0.19	82.8	.bsn
	5	NPERCP	−0.54	0.59	0	.bsn
	6	ERORGN	0.24	0.81	6.08	.hru
	7	AI1	−0.09	0.93	0.08	.wwq
	8	SOL_ORGN	−0.07	0.95	55.92	.bsn
TP	1	SOL_ORGP	19.7	0	88.3	.chm
	2	P_UPDIS	−10.62	0	13.9	.bsn
	3	PHOSKD	−6.8	0	146.1	.bsn
	4	PPERCO	2.09	0.04	16.82	.bsn
	5	AI2	−0.38	0.71	0.02	.wwq

Note: Parameters are explained in detail in Neitsch et al. [25].

**Table 3:** Guishui River Basin Data List.

Scenarios	BMPs	Practices Setting	Parameter Adjustment
S1	Structural measures	Grassed Waterway	.ops file adjusts parameters in GW
S2		VFS (5m)	.ops file FS width is set to 5 m
S3	Nonstructural measures	Fertilization reduction (10%)	.mgt file modify the amount of fertilizer
S4		Fertilization reduction (20%)	
S5		No-tillage	.mgt file to join Tillage

**Table 4:** Control unit specific information.

ID	Control Unit Name	River	Administrative District	Area (km^2^)
1	1-Gucheng River-Zhangshanying	Gucheng River	Zhangshanying	54.74
2	2-Guishui River-Zhangshanying	Guishui River	Zhangshanying	3.97
3	3-Sanli River-Zhangshanying	Sanli River	Zhangshanying	5.97
4	4-Xibazi River-Kangzhuang	Xibazi River	Kangzhuang	2.7
5	5-Xiaozhangjiakou River-Badaling	Xiaozhangjiakou River	Badaling	6.4
6	6-Xibazi River-Badaling	Xibazi River	Badaling	26.37
7	7-Guishui River-Jingzhuang	Guishui River	Jingzhuang	9.32
8	8-Guishui River-Jingzhuang	Guishui River	Jingzhuang	11.72
9	9-Baolinsi River-Jingzhuang	Baolinsi River	Jingzhuang	4.86
10	10-Xierdao River-Jingzhuang	Xierdao River	Jingzhuang	28.85
11	11-Xiaozhangjiakou River-Jingzhuang	Xiaozhangjiakou River	Jingzhuang	28.72
12	12-Wulipo River-Jiuxian	Wulipo River	Jiuxian	8.55
13	13-Xilongwan River right branch-Jiuxian	Xilongwan River right branch	Jiuxian	18.49
14	14-Xilongwan River-Jiuxian	Xilongwan River	Jiuxian	23
15	15-Guishui River-Jiuxian	Guishui River	Jiuxian	5.2
16	16-Gucheng River-Jiuxian	Gucheng River	Jiuxian	36.1
17	17-Xilongwan River-Jiuxian	Xilongwan River	Jiuxian	19.67
18	18-Guishui River-Dayushu	Guishui River	Dayushu	9.93
19	19-Guishui River-Dayushu	Guishui River	Dayushu	4.99
20	20-Xibazi River-Dayushu	Xibazi River	Dayushu	14.99
21	21-Xiaozhangjiakou River-Dayushu	Xiaozhangjiakou River	Dayushu	14.3
22	22-Guishui River-Yanqing	Guishui River	Yanqing	9.17
23	23-Sanli River-Yanqing	Sanli River	Yanqing	14.72
24	24-Guishui River-Yanqing	Guishui River	Yanqing	10.97
25	25-Guishui River-Yanqing	Guishui River	Yanqing	4.84
26	26-Guishui River-Yanqing	Guishui River	Yanqing	4.44
27	27-Xiaozhangjiakou River-Yanqing	Xiaozhangjiakou River	Yanqing	3.28
28	28-Gucheng River-Shenjiaying	Gucheng River	Shenjiaying	5.83
29	29-Guishui River-Shenjiaying	Guishui River	Shenjiaying	23.74
30	30-Guishui River-Xiangying	Guishui River	Xiangying	36.16
31	31-Guishui River-Liubinpu	Guishui River	Liubinpu	65.34
32	32-Zhoujiafen River-Yongning	Zhoujiafen River	Yongning	18.82
33	33-Sanlidun River-Yongning	Sanlidun River	Yongning	17.05
34	34-Sanlidun River-Yongning	Sanlidun River	Yongning	23.15
35	35-Guishui River-Yongning	Guishui River	Yongning	10.26
36	36-Konghuaying River-Yongning	Konghuaying River	Yongning	43.76
37	37-Guishui River-Sihai	Guishui River	Sihai	3.41
38	38-Gucheng River-Zhangshanying	Gucheng River	Zhangshanying	3.11
39	39-Wulipu River-Zhangshanying	Wulipu River	Zhangshanying	34.14
40	40-Xibazi River-Badaling	Xibazi River	Badaling	7.26
41	41-Guishui River-Yongning	Guishui River	Yongning	24.09
42	42-Guishui River-Yongning	Guishui River	Yongning	6.55
43	43-Xierdao River-Dayushu	Xierdao River	Dayushu	4.39
44	44-Guishui River-Dayushu	Guishui River	Dayushu	11.29

**Table 5:** Scenario reduction efficiency.

Scenarios	Reduction Efficient %		Average Reduction Efficiency %		Reduction Amount (kg)	
	TN	TP	TN	TP	TN	TP
S1	13.2–46.3	15.6–49.6	35.1	33.7	7408.3	2478.42
S2	33.6–76.6	43.6–74.2	63.4	62.6	13,381.38	4603.82
S3	0.3–4.1	0.7–12.9	2.4	4.4	506.55	323.59
S4	0.5–8.2	1–19.4	4.7	8	992	588.35
S5	0–0.9	0.2–6.5	0.2	0.8	42.21	58.83

**Table 6:** Set scenario costs.

Scenarios	Unit Costs (€/ha)	Implementation Area (ha)	Total Costs (1000 €)
S1	206	20	4.12
S2	615	136	83.655
S3	8	5441	43.528
S4	14	5441	76.174
S5	13.5	5441	73.454
Total	/	/	280.931
